# A holistic perspective to predict yoga tourists’ revisit intention: An integration of the TPB and ECM model

**DOI:** 10.3389/fpsyg.2022.1090579

**Published:** 2023-02-15

**Authors:** Eusebio C. Leou, Huiqing Wang

**Affiliations:** ^1^Institute of Research on Portuguese-Speaking Countries, City University of Macau, Macao, Macao SAR, China; ^2^School of International Tourism, Anhui International Studies University, Hefei, Anhui, China

**Keywords:** yoga tourism, theory of planned behavior (TPB), experience expectations, expectation confirmation model (ECM), revisit behavioral intention, revisit intention, behavior intentions

## Abstract

**Introduction:**

The purpose of this study is to empirically investigate the pattern of visitors’ revisiting behavioral intention via the innovational approach of Theory of Planned Behavior (TPB) and the Expectation Confirmation Theory (ECT).

**Methods:**

This research was conducted by data collection with structured questionnaires as its instrument, which was distributed among 420 yoga tourism visitors in two destinations, Mysore and Rishikesh in India. Collected data had been processed by confirmatory factor analysis and structural equation modeling.

**Results:**

The data analysis results showed that the behavioral attitude of yoga tourism visitors can mediate the influence of behavioral intention through the satisfaction. The findings of this study include the following points: (1) the components of attitude, subjective norm and destination image apply a direct effect on the cultural and spiritual experiences of yoga tourism visitors; (2) cultural and spiritual experiences have a direct effect on the expectation confirmation and the satisfaction of yoga tourism visitors; (3) Expectation confirmation has a direct effect on the satisfaction and the behavior intention of yoga tourism visitors; and (4) Satisfaction has a direct effect on the behavior intention of yoga tourism visitors.

**Discussion:**

This study contributed by examining the satisfaction and revisit intentions of yoga tourism visitors through an integrated study of planning behavior and expectation confirmation models, which might be refilling the scarcity of research in the tourism literature. The result of this study might offer important implications for scholars, marketers, and tourism industry to better serve this emerging niche market.

## 1. Introduction

Tourists’ intention to return reflects not only their high regard for the destination’s attractiveness but also the destination’s future economic development potential. As a result, revisiting behavior is a frequently studied construct in both industry and academia. The scholars have examined tourists’ willingness to return from a variety of perspectives, including the quality of the tourist destination experience (Jung et al., 2015; Meng and Cui, 2020), the experience of auxiliary facilities within the destination (Prentice and Hsiao, 2021), the interaction between host and visitor (Tabaeeian et al., 2022), and service quality (Tosun et al., 2015). Moreover, some scholars begin their research with tourists’ motivation (Li et al., 2010; Lee et al., 2014). From the time series perspective, the travel experience can be divided into three stages: pre-trip, on-site, and post-experience. Clearly, the majority of existing research on tourists’ revisit intentions (RI) focuses on the on-site stage, especially on the effect of the on-site experience on tourists’ future RIs.

When conducting visitor behavior research, it is critical to take a holistic approach (Leiper, 1979). According to Remoaldo and Cadima Ribeiro (2015), holistic refers to something that emphasizes the whole and its interdependence. Therefore, when considering tourists’ willingness to return, it is necessary to consider the influence and correlation among various stages of the tourism experience. Some researchers have begun to focus on how to comprehensively consider the factors of different stages in order to comprehend the behavioral intention of tourists, such as Li et al. (2020), who divided tourists’ experience into three stages: pre-trip, on-site, and post-trip in order to investigate the interaction among factors before, during, and after tea tourists travel. Li et al. (2022) diverged from conventional research by focusing on tourists’ sharing behavior before travel and its impact on their subsequent travel itinerary and travel experience, rather than sharing behavior during or after travel. The inner mechanisms underlying tourist behavior have been thoroughly elucidated by previous research.

Existing research on tourist revisit behavior generally begins with the experience or experience quality during the tourism process and investigates how it influences the tourists’ intention to return. Few scholars have also considered and incorporated pre-travel stage factors into the framework of their research. In order to predict tourists’ intentions to return, this study will combine the theory of planned behavior (TPB) and the expectation confirmation theory (ECT) to develop a comprehensive theoretical model.

Participants in yoga tourism who traveled to India were the focus of this study. The reasons listed below explain why this particular group was chosen. Yoga tourism was selected as the focus of the study due to its uniqueness and rapid growth. Yoga tourism is a self-discovery journey that has the potential to transform a person’s physical, psychological, spiritual, and social consciousness. They collaborate to unite the mind, body, and spirit (Kelly and Smith, 2009). Yoga tourism is a subset of special interest and wellness tourism (Smith and Kelly, 2006), and its popularity has increased globally in recent years (Sharma and Nayak, 2018), but there is a lack of research on the topic (Cheer et al., 2017). Not only for yoga tourism managers but also for managers of other similar wellbeing tourism, it is of great theoretical and practical importance to investigate the internal mechanism that causes such tourists to return. Due to the integration of the TPB and the ECT, a second significant theoretical contribution of this study is a better understanding of the connotation of yoga tourists’ experience expectations for yoga tourism in India, as well as the factors that influence yoga tourism experience expectations. It is evident that this research will provide a solid theoretical foundation for future yoga tourism researchers.

In summary, this study raises the following three important research questions: (1) What do Indian yoga tourists expect from yoga tourism? (2) What factors influence the formation of experience expectations among yoga tourists? (3) Discuss the revisit behavior of yoga tourists and provide management enlightenment for the development of yoga tourism from the perspective of before, during, and after tourism. In the subsequent content, this research will be subdivided into two sub-studies, the relevant literature will be analyzed, and a research model will be developed, beginning with the theoretical foundation of the research.

## 2. Theoretical foundation and hypotheses development

Ajzen and Fishbein (1975, 1980) proposed the “Theory of Reasoned Action” (TRA) as a theoretical model for understanding human behavior and psychology. The TRA and its extension, the TPB, are cognitive theories that offer a conceptual framework for understanding human behavior in specific contexts. Ajzen also demonstrated the appliance of the TPB in leisure and tourism studies (Ajzen and Driver, 1992), in which the results show that after increasing the control of perceived behavior, the theoretical model of planned behavior showed the effect of enhancing the predictive ability. Ajzen explained that leisure benefit is the concept of benefit goal that the consumers expect and participate in tourism, and consumers’ expected psychology is more important than the actual perceived benefit. Using the TPB model, it would be helpful to discover the issues of the travel decision-making process. Since the TPB is a rational decision-making model, which has traditionally been used to analyze the decision-making process in many different fields, including tourism (Hamid and Isa, 2015). Actually, the visitors’ intention before travel is also an interpretation and prediction of individual willingness and behavior, which is affected by behavioral attitudes, subjective norms (SNM), and perceived behaviors. Based on the TPB, scholars analyze the visitors’ behavior and deduce that visitors’ behavior will be affected by the attitude of visitors themselves, and the attitude of visitors will affect the visitors’ behavior intention (Taylor and Todd, 1995). Sparks used the TPB model to see whether wine tourism visitors would consider another wine trip within a year of their last visit, by dividing attitude into emotional attitude and attitude toward past wine holidays. The results showed that, apart from emotional attitude, other TPB factors had a direct effect (Sparks, 2007). In general, from most tourism studies using the TPB model, the results lead that the attitudes of tourists in their pre-trip period would affect their on-site behavior intentions.

On the other hand, the TPB model has been widely employed in tourism-related studies of tourist perceptions of one-time visitors, or single-visit tourists, to a particular destination (Bianchi et al., 2017; Soliman, 2019; Hasan et al., 2020). By the current limited study about the revisit behavior by extending the TPB model, the common comments figured out that certain variables may significantly affect the RI of visitors, for instance, satisfaction, destination image (DIM), and perceived value. Visitors’ satisfaction gathered on the previous trip was confirmed as the key mediator to their RI (Abbasi et al., 2021). In another word, visitors’ attitude before their revisit (pre-visit), such as the satisfaction from their previous visit, is the determining factor of their RI. Under this tone, it seems that the pre-visit attitude would determine the RI. However, the mentioned approach seems to ignore the re-visitors’ previous experience earned from their first visit to the same destination. From the current literature about revisit behavior, it is mostly considered that the decision-making of revisit and its behavior are the results of previous experience (Adongo et al., 2015; Kim et al., 2021). In fact, there is no revisit study that has yet been used concerning the pre-visit factors. Since the TPB model has been extended in tourism-related studies of visitors’ behavior, this study attempts to extend the TPB model to discuss how the attitude, perceived behavior control, and SNM affect the revisiting behavior of yoga tourism visitors.

Obviously, tourism is a holistic behavior of visitors, which consists of three stages: pre-trip, on-site, and post-experience. It is necessary to consider the entire stages of the tourists’ behavior as a holistic body when we examine the revisit behavior of tourists to a destination. Previous research on destination loyalty shows that one of the most decisive factors for encouraging tourists’ revisiting to a destination is the visitors’ previous satisfaction or dissatisfaction with their previous stays (Appiah-Adu et al., 2000; Bigne et al., 2001; Yoon and Uysal, 2005; Alegre and Garau, 2010; Prayag and Ryan, 2012; Eusébio and Vieira, 2013). Including satisfaction or dissatisfaction, the experience of tourists from their previous visit to a destination would be one of the key factors to affect the pre-trip attitude of their revisiting the same destination (Hasan et al., 2020).

As RI is related to psychological and behavioral mechanisms, it becomes an emerging topic for scholars in tourism research. In order to clarify the relationships between the destination and the antecedents of RI, most of the mentioned studies are based on the factors such as DIM (Marine-Roig and Huertas, 2020; Rasoolimanesh et al., 2021), destination attachment (Isa et al., 2020; Cho, 2021), destination branding (Gupta et al., 2022; Rahman et al., 2022), destination authenticity (Loureiro, 2020; Kumar et al., 2022), destination personality (Jahandide Topraghlou et al., 2020; Yang et al., 2021a), and destination and visitors’ behavior (Abbasi et al., 2021; Šegota et al., 2022). By the repetition in visiting, visitors demonstrate their loyalty to a destination, which becomes support to the sustainability of tourism development (López-Sanz et al., 2021). However, the current study on the mentioned dimension can be insufficient.

Currently, the existing research is more likely to agree that the revisiting behavior of visitors can be considered as the result of the post-experience. It seems more necessary to provide further study by a holistic approach to the factors about the periods of pre-trip, on-site, and post-experience. To reach the mentioned goal, the authors attempt to clarify the impact of prior experience to revisit behavior, by applying the approach of ECT. According to the perspective of ECT, customers’ repurchase intention is determined by their satisfaction with the previous use of the product or service (Oliver, 1980). From this concept, in the context of tourism, the attitude of revisit tourists would be significantly different from the attitude of one-time visitors since the visitors’ experience gained from prior or first-time visits might be a factor to affect their attitude toward revisit behavior.

Regarding culture tourism, the relationship among destination personality, self-congruity, and RI is critical to understanding how tourists decide to revisit a destination (Yang et al., 2021b). Exotic culture is defined as culture shock (Yang et al., 2013). Cultural distance is one of the main mechanisms related to marketing outcomes, especially in terms of tourists’ RIs, which has the potential to exacerbate misunderstandings between hosts and guests as they interpret cues differently (Reisinger and Turner, 2003; Vieira et al., 2021), leading to communication barriers. Tourists’ perception of the external environment may increase their anxiety and uncertainty (Chen et al., 2011; Yang et al., 2021a). Hofstede’s argument is that uncertainty avoidance is more important than any other cultural dimension in predicting travel behavior from a cross-cultural perspective. Uncertainty avoidance is interpreted as the degree to which society perceives itself to be intimidated by any uncertain and ambiguous situation and tries to avoid it. By adopting the cultural dimension approach of Hofstede, it can be seen that the loyalty of visitors across cultures may be predicted by individualism and uncertainty avoidance, and their behavior intention might be evaluated as a determination of tourism internationalization (Yang et al., 2022b).

Many studies explore a destination’s performance by analyzing declared visitor attitude with different aspects of the destination, whether satisfaction or dissatisfaction (Oliver, 1993; Soscia, 2007; Alegre and Garau, 2010; Škoriæ et al., 2021). Some studies talked about the relationship between tourists’ image perception of a tourist destination and a tourist product and their experience expectations (Sharma and Nayak, 2019; Pimtong et al., 2021; Ajay et al., 2022; Carreira et al., 2022; Karakaş et al., 2022; Nautiyal et al., 2022). Based on self-congruity, destination personality is linked indirectly and positively with visitors’ revisit and recommended behavioral intention (Yang et al., 2020). Gender factors might have direct significance among DIM, destination personality, self-congruity, and RI (Yang et al., 2021b). Actual and ideal self-congruity are the mediators among destination personality, DIM, and RI (Yang et al., 2022a). By reviewing the current literature in tourism studies, it is rare to see the application of the decision-making behavior of visitors and the revisit decision-making together with the ECT.

According to the assumption of rational human behavior, there should be a rational decision-making process for the decision-making of visiting behavior, as well as for the decision-making of revisiting behavior. The essence of tourism is an experience (Grappi and Montanari, 2011; Prayag and Ryan, 2012; Prayag et al., 2013; Boo and Busser, 2018; Sharma and Nayak, 2019), which can meet certain expectations of tourists. Although the ECT has been used in the technical field in the past (Oliver, 1980; Anderson and Sullivan, 1993; Patterson and Spreng, 1997; Dabholkar et al., 2000), the ECT can also be one of the angles to explain the rational process of tourist behavior (Bem, 1972; Prayag et al., 2013). However, from the limited studies on RI by the application of the ECT, it was clarifying the increase in visitors’ social return (SR) effects the visit intention to the same destination (Sedera et al., 2017). From the ECT, it is demonstrated the impacts between SR and memorable tourism experience (MTE), as well as the weak link of SR on RI (Mittal et al., 2022).

This study aims to develop an empirical framework to discover the existing black box, in which we would like to demonstrate the key factor of the previous experience earned from their first visit to a certain destination, in affecting the pre-trip attitude. To this end, ECT would be adopted to explain the rational process of visitors’ behavior. In this study, the authors try to adopt the application of ECT to explain the process of yoga tourism visitors’ experience and the process of visitors’ rational decision-making behavior. It would be an innovational application of the ECT, which may verify the expectancy behavior in the pre-trip period, the experience expectation confirmation in the on-site period, and the satisfaction in the post-experience period.

Based upon the previous extensive theoretical foundation, a framework is proposed and constructed accordingly to evaluate the experiential expectation of yoga tourism visitors. There are eight research components in total, including attitude (ATT), SNM, DIM, cultural experience-based on norm expectations (EPf), spiritual experience-based on norm expectations (EPs), expectation confirmation (CFM), satisfaction (SAT), and RI. In view of the specific research objective of this study, attitude, subjective norm, and destination image are deemed as exogenous variable components, while cultural and spiritual experiences, expectation confirmation, satisfaction, and RIs are designated as endogenous variables. Meanwhile, in the consideration of the characteristics of all the research components, these are all deemed latent variables as measured by other observable variables.

**FIGURE 1:**
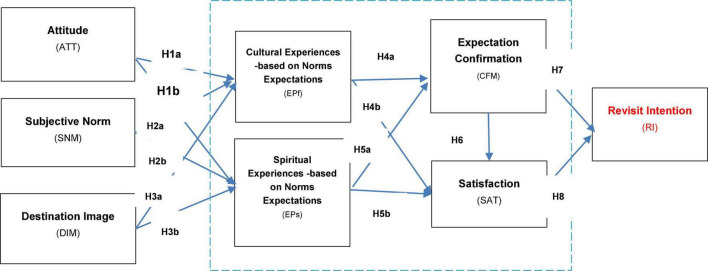
Research model and hypothesis of the study.

The interrelationship among the research components in this study is constructed as below: (1) attitude has a direct effect on the cultural and spiritual experiences of yoga tourism visitors; (2) Subjective norm has a direct effect on the cultural and spiritual experiences of yoga tourism visitors; (3) Destination image has a direct effect on the cultural and spiritual experiences of yoga tourism visitors; (4) cultural and spiritual experiences have a direct effect on the expectation confirmation of yoga tourism visitors; (5) cultural and spiritual experiences have a direct effect on the satisfaction of yoga tourism visitors; (6) expectation confirmation has a direct effect on the satisfaction of yoga tourism visitors; (7) expectation confirmation has a direct effect on the RIs of yoga tourism visitors; and (8) satisfaction has a direct effect on the RIs of yoga tourism visitors. In this sense, expectation confirmation has a moderating effect on the relationship between spiritual experiences and satisfaction. In addition, a partial mediating function is performed by satisfaction between expectation confirmation and RIs (refer to Figure 1).

Most previous research has focused on the relationship among experience quality, visitor satisfaction, and behavior intentions (Muhammad et al., 2021). In fact, the quality and content of experience vary not only among customers but also among employees, and the connotation of experience quality varies substantially across industries (Yang et al., 2015). Despite its significance as a component of wellness tourism, the public lacks a comprehensive understanding of the connotation of yoga tourism, particularly yoga tourism in India. Few scholars in the existing literature, including Aggarwal et al. (2008), Ali-Knight and Ensor (2017), and Sharma and Nayak (2019) have conducted an initial discussion on the connotation of the yoga tourism experience. Therefore, a more in-depth discussion of the experience connotation of yoga tourists to India will aid academics and the yoga tourism industry in gaining a more comprehensive understanding of yoga tourism. Similarly, the Service Quality Gap Theory (Parasuraman et al., 1985) recommends that the understanding of experience quality be thoroughly examined from the perspectives of expectations and actual performance. The study of experience necessitates not only the measurement of actual perception, but also an appreciation of experience expectations (Hou et al., 2020). Keeping in mind the aforementioned justification, this study will employ the methods used to develop the scale in order to investigate the experience expectations of yoga tourists visiting India.

## 3. Methodology and research design

### 3.1. Study site and context

Yoga is deeply embedded in Indian culture, history, and society and is considered a symbol of Indian cultural identity. The origin of yoga can be traced back to the ancient Indus Valley Civilization (Harappa), which is about 5,000 years ago (Dhyansky, 1987). For the Western audience, yoga was initially considered a practice of the ancient Hindu religious component. Through yoga techniques, people believe that they have changed their lifestyle. Yoga is not only a heritage of India but also has its influence in different countries (Cheer et al., 2017). In Resolution 69/131 adopted by the United Nations on 11 December 2014, it was announced that June 21 was designated as International Yoga Day, which aims to raise the world’s awareness of the many benefits of yoga practice. On 1 December 2016, the UNESCO Intergovernmental Committee for the Protection of Intangible Cultural Heritage (ICH) passed a resolution (ITH/16/11.COM/10.b), including Indian Yoga as item No. 01163 in the ICH List of Humanity. Recognizing that yoga has a wide range of attractiveness with its wellness function for healthcare, yoga practice later spreads in various forms all over the world and becomes more and more popular (Agoramoorthy, 2019).

With its unique universal phenomenon integrating religions, traditions, cultures, color, and nation, yoga tourism is a subset of wellness tourism nurtured across the world, which provide visitors from other countries a reason the “travel to feel well” (Charak et al., 2021). As an indigenous therapy that originated in India, yoga becomes an attraction for visitors, particularly for those yoga fans who are fascinated by this wellness practice and philosophy experiences. Yoga fans visit India to immerse themselves in yoga tourism trusting the authenticity of the country (Rungsimanop and Ashton, 2021). The authenticity of yoga seems to be a reason for yoga fans to their visit and RI to India. India received 17.9 million international arrival visitors in 2019, including many visitors seeking a yoga experience. Before the COVID-19 pandemic, the yoga tourism sector generated around USD 6.5 billion yearly (Kathuria, 2020). In the Indian State of Uttarakhand, yoga tourism investment is projected to reach USD 85 billion by 2028, which is over 50% growth forecast reliant on international arrival visitors (McCartney, 2021). However, from the Spring of 2020, yoga tourism went to a standstill ever since the global tourism sector was lockdown by the travel restrictions caused by the COVID-19 pandemic (Anand, 2021).

As wellness and spiritual needs are the major desires for yoga-based visitors to India, yoga tourism is an emerging market in India, with great economic potential element in wellness sectors to boost revenue in the tourism and hospitality industries (Dayananda Swamy and Agoramoorthy, 2021). In a related study of yoga tourism in India, it has been explored that destination attractiveness and destination uniqueness were found as significant attributes of visitor experience satisfaction (Singh and Dhakhar, 2022). Due to the historical background of yoga, India being a hub of yoga offers a variety of destinations. In India, there are some yoga-based tourism hotspots, such as Mysore (the origin of Ashtanga Yoga), Bangalore, Goa, Trivandrum, Pune, and Rishikesh (the origin of Indian Yoga) (Ranjan et al., 2022). Considering the geographical location and iconic landscapes, the field investigation of this study was conducted in Mysore and Rishikesh.

### 3.2. Measurement

This study employs a mixed research approach, with the overall investigation divided into two sub-studies and conducted gradually. The first sub-study aims to establish the connotation of yoga tourists’ experience expectations. This sub-study utilized the methodology proposed by Churchill (1979) and Tsaur et al. (2010). Initially, from June to August 2019, interviews were conducted in China using a snowball sampling technique with individuals who had visited India for yoga tourism the previous year in order to better understand the respondents’ expectations for yoga tourism in India. Between the 26th and 30th interviewees, no new information was found. Consequently, according to this study, the interview data have reached theoretical saturation at this point.

Based on the aforementioned interviews and a few bodies of literature relating to yoga tourism, this study clarifies the expected item bank of yoga tourism experiences. From September to November 2019, the first round of questionnaires was administered on the social platform of yoga enthusiasts, and exploratory factor analysis (EFA) was performed on the data to determine the fundamental framework of tourists’ yoga tourism experiences. The researchers then traveled to India in January 2020 to administer a second round of data collection questionnaires. The investigation was conducted in Mysore and Rishikesh. Before distribution, the questionnaire was validated for tourists’ purposes. Once confirming the visitors’ purpose to India is for yoga practice, the questionnaires were interpreted and distributed. A non-random sampling method was used, and this study successfully surveyed 402 respondents who were eligible and willing to participate in the survey. To facilitate study 2, that is to build up a more comprehensive interpretation of yoga tourists’ RI based on the integration of TPB and ECM, the second round of questionnaires distributed in India included additional constructs associated with this sub-study. The second sub-study aims to build an integrated TPB and ECM model. This sub-study employed quantitative research methods to validate the previously developed research model and hypotheses, using the aforementioned questionnaire data collected in India.

## 4. Results

The analytical technique conducted in the main survey of this research is structural equation modeling (SEM) analysis *via* AMOS 24 software. The first stage is the study of the measurement model, which is used to evaluate whether the measured variables can measure their latent variables correctly in the model. The second stage is the study of the structural model, which analyzes the effect and explanatory power of causality using the structural model (Anderson and Gerbing, 1988).

### 4.1. The connotation of expected yoga tourism experience

Based on a theoretical foundation and interviews, this study categorized 18 items related to the experience expectations of yoga tourists, leaving 11 items after screening by three experts. Through the EFA of 253 valid data obtained from the first round of questionnaires, the results indicated that the first and third items were deleted because the community’s extraction was less than 0.5, and the remaining nine items were subsequently analyzed by the EFA and could be divided into two groups. The content summarizes two dimensions the expectation of functional experience (EPC2, EPC4, EPC5, and EPC6) and the expectation of emotional experience (EPC7, EPC8, EPC9, EPC10, and EPC11). Specific information is provided in Table 1.

**TABLE 1:** Rotated component matrix^a^ (*n* = 253).

	Component	
	**Functional**	**Emotional**
EPC2		0.738
EPC4		0.732
EPC5		0.848
EPC6		0.694
EPC7	0.777	
EPC8	0.781	
EPC9	0.831	
EPC10	0.845	
EPC11	0.729	

Extraction method: Principal component analysis. Rotation Method: Varimax with Kaiser Normalization.

^a^Rotation converged in three iterations.

### 4.2. Measurement model

The software AMOS 24 is used for the convergence validity and discriminant validity analysis. The convergence validity test is tested by the Standardized Estimate (S.E.), Composite Reliability (CR), and Average Variance Extracted (AVE) of the measurement entry. The results suggest that the number of the remaining latent variables is greater than 0.7, with an exception for the sense of disgust, the number is slightly lower than 0.7. Besides, in case of the exception for the sense of disgust and deprivation, the number of other latent valuables is larger than 0.5. From the specific results of the Confirmatory Factor Analysis (CFA) are demonstrated as Table 2, it indicates that the data has good convergence validity (Table 2, CFA significant Confirmatory Factor Analysis).

**TABLE 2:** Confirmatory Factor Analysis (CFA) of expected experience.

			Estimate	S.E.	*t*-value	CR	AVE	Cronbach α
EPC2	< —	EPf	0.678			0.824	0.539	0.829
EPC4	< —	EPf	0.771	0.121	12.927			
EPC5	< —	EPf	0.776	0.126	12.981			
EPC6	< —	EPf	0.708	0.104	12.090			
EPC7	< —	EPs	0.672			0.877	0.589	0.882
EPC8	< —	EPs	0.800	0.074	13.917			
EPC9	< —	EPs	0.755	0.068	13.265			
EPC10	< —	EPs	0.803	0.072	13.950			
EPC11	< —	EPs	0.799	0.075	13.897			

Table 3 is a matrix of correlation coefficients, showing that fit also meets the criteria of the Confirmatory Factor Analysis (CFA) (Hu and Bentler, 1999) (Table 3, CFA significant Confirmatory Factor Analysis).

**TABLE 3:** Confirmatory Factor Analysis (CFA) fitness coefficient.

Measure	Estimate CFA	Estimate SEM	Threshold	Interpretation
CMIN/DF	2.032	2.133	Between 1 and 3	Excellent
CFI	0.934	0.942	>0.95	Acceptable
RMSEA	0.051	0.053	<0.06	Excellent
PClose	0.394	0.205	>0.05	Excellent

The results of the Confirmatory Factor Analysis (CFA) are shown in Table 4. The factors loading or SMC and AVE values of items in every measurement aspect all comply with the reliability test standards (Hair et al., 2010). Table 5 is a matrix of correlation coefficients and discriminative validity coefficients, showing that discriminative validity also meets the standard (Bagozzi, 1981) (Table 4, CFA significant Confirmatory Factor Analysis).

**TABLE 4:** Confirmatory Factor Analysis (CFA) analysis results.

			Estimate	S.E.	*t*-value	CR	AVE	Cronbach α
ATT1	< —	ATT	0.732			0.888	0.570	0.890
ATT2	< —	ATT	0.792	0.078	15.391			
ATT3	< —	ATT	0.783	0.076	15.214			
ATT4	< —	ATT	0.766	0.087	14.879			
ATT5	< —	ATT	0.721	0.085	13.984			
ATT6	< —	ATT	0.733	0.080	14.205			
SNM1	< —	SNM	0.721			0.744	0.500	0.763
SNM2	< —	SNM	0.783	0.098	10.058			
SNM3	< —	SNM	0.593	0.076	9.561			
PBC1	< —	PBC	0.700			0.808	0.587	0.856
PBC2	< —	PBC	0.896	0.083	12.754			
PBC3	< —	PBC	0.685	0.079	12.215			
CFM1	< —	CFM	0.847			0.855	0.664	0.859
CFM2	< —	CFM	0.820	0.056	18.630			
CFM3	< —	CFM	0.775	0.044	17.329			
SAT1	< —	SAT	0.785			0.868	0.688	0.873
SAT2	< —	SAT	0.863	0.051	18.499			
SAT3	< —	SAT	0.838	0.051	17.926			
EPC2	< —	EPf	0.678			0.824	0.539	0.829
EPC4	< —	EPf	0.771	0.121	12.927			
EPC5	< —	EPf	0.776	0.126	12.981			
EPC6	< —	EPf	0.708	0.104	12.090			
EPC7	< —	EPs	0.672			0.877	0.589	0.882
EPC8	< —	EPs	0.800	0.074	13.917			
EPC9	< —	EPs	0.755	0.068	13.265			
EPC10	< —	EPs	0.803	0.072	13.950			
EPC11	< —	EPs	0.799	0.075	13.897			
RI1	< —	RI	0.745			0.871	0.693	0.892
RI2	< —	RI	0.907	0.065	17.606			
RI3	< —	RI	0.838	0.063	16.668			
DIM1	< —	DIM	0.817			0.711	0.554	0.736
DIM2	< —	DIM	0.664	0.105	9.489			

**TABLE 5:** Discriminant validity.

	ATT	SNM	PBC	CFM	SAT	EPf	EPs	RI	DIM
ATT	**0**.**755**								
SNM	0.124*	**0**.**707**							
PBC	0.142*	0.159*	**0**.**766**						
CFM	0.501***	0.118†	0.227***	**0**.**815**					
SAT	0.600***	0.098	0.306***	0.807***	**0**.**829**				
EPf	0.405***	0.145*	0.285***	0.464***	0.403***	**0**.**734**			
EPs	0.388***	0.128*	0.218***	0.519***	0.430***	0.650***	**0**.**767**		
RI	0.437***	0.242***	0.291***	0.562***	0.630***	0.478***	0.521***	**0**.**833**	
DIM	0.618***	0.247***	0.095	0.344***	0.400***	0.226***	0.259***	0.424***	**0**.**744**

*Significant in 0.05 level; ***significant in 0.001 level; and ^†^significant 0.000 level. Bold values significant the squared root of AVE.

### 4.3. Path analysis

Through the application of the software AMOS 24, path analysis of latent variables can be carried out with the involved structural model. The path analysis results are demonstrated in Table 6. Attitude has a significant positive impact on the cultural and spiritual experiences (β = 0.455 and 0.414, *p* < 0.001), indicating that the higher the attitude evaluation of yoga tourism visitors, the higher the cultural and spiritual experiences they receive. In contrast, the subjective norm has not had a significant positive impact on the cultural and spiritual experiences (β = 0.117 and 0.086, *p* = 0.063 and 0.158), and the destination image has not had a significant positive impact on the cultural and spiritual experiences (β = −0.078 and 0.016, *p* = 0.387 and 0.859), indicating that both the subjective norm and destination image evaluation of yoga tourism visitors are not significant with the cultural and spiritual experiences they receive.

**TABLE 6:** Path analysis results.

Path (hypothesis)			Estimate	S.E.	*t*-value	*P*	Support
EPf	< —	ATT	0.455	0.105	5.151	***	Yes
EPs	< —	ATT	0.414	0.146	4.969	***	Yes
EPf	< —	SNM	0.117	0.028	1.856	0.063	No
EPs	< —	SNM	0.086	0.04	1.413	0.158	No
EPf	< —	DIM	-0.078	0.059	-0.865	0.387	No
EPs	< —	DIM	0.016	0.084	0.178	0.859	No
CFM	< —	EPs	0.423	0.091	5.255	***	Yes
CFM	< —	EPf	0.171	0.135	2.139	0.032	Yes
SAT	< —	EPs	0.051	0.064	0.688	0.491	No
SAT	< —	EPf	0.082	0.089	1.169	0.242	No
SAT	< —	CFM	0.677	0.049	10.362	***	Yes
RI	< —	SAT	0.442	0.098	5.088	***	Yes
RI	< —	CFM	0.230	0.072	2.747	0.006	Yes

***Significant in 0.001 level.

Cultural and spiritual experiences have a significant positive impact on the expectation confirmation (β = 0.423 and 0.171, *p* < 0.001 and *p* = 0.032), indicating that the higher the cultural and spiritual experience evaluation of yoga tourism visitors, the higher the expectation confirmation they receive. On the other hand, cultural and spiritual experiences do not have a significant positive impact on satisfaction (β = 0.051 and 0.082, *p* = 0.491 and 0.242), indicating that the higher cultural and spiritual experience evaluation of yoga tourism visitors is not significant with the satisfaction they receive.

Expectation confirmation has a significant positive impact on satisfaction (β = 0.677, *p* < 0.001), indicating that the higher the expectation confirmation evaluation of yoga tourism visitors, the higher the satisfaction they receive. Satisfaction has a significant positive impact on the RIs (β = 0.442, *p* < 0.001), indicating that the higher the satisfaction evaluation of yoga tourism visitors, the higher the RIs they receive. Expectation confirmation has a significant positive impact on the RIs (β = 0.230, *p* = 0.006), indicating that the higher the expectation confirmation evaluation of yoga tourism visitors, the higher the RIs they receive.

## 5. Discussion and conclusion

This study is one of the few studies that explore the experience expectations of yoga tourists. Based on the following process of developing the scale, it can be concluded, from the above study, that the expectations of yoga tourism visitors in India would be figured out as two aspects: functional expectation experience and emotionally expected experience. Meanwhile, by creative integration of the TPB theory and the ECM model within the mentioned study, it would be helpful for better comprehension of the observation to the revisit behavioral intention of yoga tourism visitors. Moreover, the most meaningful contribution of this study can be considered as the exploration of the unexplained gray zone of the TPB model, which has been in place for years, about the interrelationship between attitude and revisit behavioral intention. After a series of empirical research method in this current study, it can be demonstrated that human behavioral intentions can be understood better through the concept integration of two models, the ECM and the TPB.

### 5.1. Theoretical implications

The findings of this study reveal several intriguing findings. First, the experience expectations of yoga tourism visitors can be divided into two groups: functional experience expectations and spiritual experience expectations. According to the interviews conducted with the respondents of this study, the so-called functional experience expectation refers primarily to the hope that the specific movements of yoga can be enhanced through the yoga experience in India, such as learning more authentic or advanced physical postures, breathing techniques, and body control. As a result of their trip to India, participants are anticipated to gain spiritual relaxation or enlightenment experiences, such as meditation or mind control. Although yoga tourists’ experience expectations are consistent with Ahn and Picard’s (2014) two dimensions of experience, namely, affective experience and cognitive experience, the expectations of yoga tourists are unique. Regarding the anticipated content of the particular experience, additional research is required based on alternative tourism markets. For instance, Öznalbant and Alvarez (2020) divided yoga tourism into three categories: yoga-focused, cultural tourism-focused, and wellness-focused. Clearly, wellness tourism research requires in-depth and targeted investigation. This is the only way to gain a more specific and targeted understanding of tourists’ expectations regarding their tourism experience.

In addition, the TPB theory is incorporated into this research in terms of the factors that influence experience expectations. This study introduced pre-travel images of India as a tourist destination, along with the concepts such as yoga attitudes and subjective behavioral norms. It is expected to provide a more thorough description of the potential factors that influence tourists’ decisions to revisit India. The findings of the study indicated that subjective norms and perceptions of the destination’s image have no significant effect on yoga tourism visitors’ experience expectations. The anticipated experience was significantly influenced solely by one’s attitude toward yoga. In other words, an individual’s inner attitude toward yoga has a larger influence on his or her yoga tourism. This occurs frequently within the yoga community. According to Grover et al. (1987), people’s attitudes toward yoga impact its effects. Moreover, Venkatesh et al. (1994) discovered that yoga practitioners have a significantly more positive opinion of yoga than non-yoga practitioners. This could be due to the distinctive characteristics of yoga tourism products. If a different type of wellness tourism is substituted for the study’s location, the findings of the study may change. Future studies could examine the research framework proposed in this study from a variety of perspectives.

Despite the fact that the attitude toward yoga is more focused on generating functional experience expectations, the path coefficient and *t* value are greater in terms of spiritual experience expectations in terms of expectation confirmation impact factors. Given that the primary internal logic of this research model is to construct a set of holistic explanations for yoga tourists’ return behavior, the aforementioned conclusions may reveal the transformative power of yoga tourism (Ponder and Holladay, 2013) from a different perspective. Thus, yoga tourists visiting India are initially impacted by their yoga attitudes. Initially, they expected to be able to learn yoga skills from behavior, but during the yoga tourism experience, the expectation of spiritual experience has a greater influence on the confirmation of tourist experience expectations than the expectation of functional experience. In addition, Bowers and Cheer (2017) research on yoga tourism supports the aforementioned viewpoint. All of the above-mentioned scholars acknowledged the transformative power of yoga tourism, but they all explained it in terms of logical deduction. This is the first study to use large sample data collection and empirical analysis to illustrate the transformation process of tourists in the specific model path coefficient, demonstrating that the combination of TBP and ECT would contribute to the existing body of tourism knowledge.

In summary, the finding of the current study demonstrated that expectation confirmation mainly affects satisfaction. Although it also affects revisit behavioral intention, it affects behavior more through the transmission mechanism of satisfaction. It is actually consistent with the theoretical foundation of the hypotheses proposed in this study, the Service Quality Gap Theory (Parasuraman et al., 1985). Only if the expectations are consistent with the actual experience, people will be satisfied and have the revisit willingness. The merely experience expectations do not directly affect consumers’ satisfaction.

### 5.2. Management implications

In order to explore the unexplained gray zone about the interrelationship between attitude and revisit behavioral intention that was originally ignored, this study combines the TPB and the ECM models and provides a new explanation for understanding the theoretical connotation of TPB. The elements of different stages of tourism, such as subjective norm (pre-visit), destination image (pre-visit), experience expectation (pre-visit), experience confirmation (on-visit), satisfaction (post-visit), and revisit behavior intention (post-visit) can be integrated into one single model. This integrated model can be applied to revisit behavioral study, which can provide a more systematic and holistic understanding of revisit behavior.

From the above study, it can be found that the influence of attitude on functional experience expectations is slightly higher than the influence of emotional experience expectations, which also reflects that yoga tourism is relatively special in contrast to other tourism experiences. At the very beginning of planning and forming expectations before the trip, specific functional expectations are the domination factors for the decision-making. During the evaluation and decision period for RIs, the emotional experience confirmation plays a role as an even more important influencing factor. This seemingly contradictory result just confirms that finding from the on-site interview in India, many yoga tourism respondents expressed that they felt more relaxed after arriving in India. For these yoga tourism visitors, perhaps, one of the initial purposes of going to India was to have more professional and authentic Yoga practice; however, after a period of immersion and washing in India, their attitude and understanding of yoga have been upgraded.

This research also has certain enlightenment value for the promotion and development of yoga tourism. First, the study carefully discusses the experience of yoga tourism visitors and comes to a relatively stable and reliable conclusion. It is to say that yoga tourism visitors will improve their practice skills on one hand and experience the culture and atmosphere of yoga on the other hand, since these are two key expectations for yoga tourism in India. As the yoga tourism destination, for Mysore and Rishikesh in India, it is more likely to focus on destination promotion as well as destination image branding issues. However, it is necessary to classify the visitors upon their visit history, since the visit behavioral intention of the newcomer visitors would vary from those repeat visitors and their revisit behavioral intention. From the result of the interview, the newcomer visitors may be dominated by utilitarian expectations, but when they decide to visit again the same destination, they become repeat visitors and would be dominated by spiritual or emotional expectation confirmation as their prerequisite. For this reason, the authority of the destination seems to adopt different strategies for newcomer and repeat visitors. In this case, for the authority as well as the industries of the yoga tourism destination, such as the testing sites of this study Mysore and Rishikesh in India, it seems to adopt more actions to consolidate the loyalty of yoga visitors.

### 5.3. Research limitations and future research prospects

Mixed research of qualitative and quantitative methods was adopted in this current study, and consequently, the obtained results seem relatively valuable. However, certain research limitations do exist. The qualitative research phase of this study was mainly conducted in mainland China. Considering that respondents from different cultural backgrounds may have different experience expectations, the framework of the current yoga tourism experience expectations might be verified by extending to a broader context of cultural background. Besides, most of the sampling methods used in this study are non-probability sampling. For the upcoming future, the sampling methods seem to be optimized to obtain more representative samples, which would be more conducive to the stability of the research conclusions, since the non-random sample has to be treated with great care. Furthermore, from the conceptual/contextual point of view, the RI may be applicable to further research for alternative special interest tourism (SIT), such as wine tourism (Back et al., 2021), coffee tourism (Chen et al., 2021), gastronomy tourism (Widjaja et al., 2020), astrotourism or stargazing tourism (Li, 2021; Rodrigues et al., 2022), volunteer trip (Manosuthi et al., 2020), and suicide travel (Yu et al., 2020; Yang et al., 2022c).

## Data availability statement

The original contributions presented in the study are included in the article/supplementary material, further inquiries can be directed to the corresponding authors.

## Ethics statement

Ethical review and approval was not required for the study on human participants in accordance with the local legislation and institutional requirements. Written informed consent from the participants was not required to participate in this study in accordance with the national legislation and the institutional requirements.

## Author contributions

All authors listed have made a substantial, direct, and intellectual contribution to the work, and approved it for publication.
